# A Framework for Error Correction Under Prediction

**DOI:** 10.3389/fpsyg.2012.00411

**Published:** 2012-10-17

**Authors:** Russ McBride

**Affiliations:** ^1^University of UtahSalt Lake City, UT, USA

Clark describes a budding trend in neuroscience toward what he calls a “predictive processing” model, the core idea of which is pretty simple. The brain is a prediction machine. It generates top-down predictions about expected sensory input. These collide with actual bottom-up sensory input, canceling out the matches, and leaving only the prediction errors to propagate forward. This model nicely explains a variety of otherwise anomalous experimental results and dovetails cleanly with Bayesian modeling formalisms. As such it is a wonderfully concise model that gets at a critical and under-appreciated component of brain activity – error correction under prediction.

Clark has got a hold of the steering wheel here but does not notice he is in a car… and steering wheels abound. He sees error correction under prediction everywhere he looks (a generalization I applaud), first seeing it in action in addition to sensation, then so ubiquitous as to be the unifying architecture of “mind, brain, and action” that can illuminate perception, action, learning, attention (p. 60), and potentially robust conscious experience (p. 48). The now more-broadly named “action-oriented predictive processing model” offers “the best clue yet to the shape of a unified science of mind and action” (p. 1) and promises “to bring cognition, perception, action, and attention together within a common framework” (p. 30). This is bold. This approach is not a small component of a broader theory; error correction under prediction is the general unifying theory, one that is on the verge of unveiling nothing less than an expansive, unifying account of mind, and behavior.

As such it is false, and it is false for an important reason – we are not essentially prediction machines – and so, as a general unifying account of mind and action it is radically misguided. But it is also correct for important reasons. Error correction under prediction *is* everywhere. The question is, why? This question is, perhaps, the single most important question to ask about mind and behavior, and it has a simple answer, one which I shall try to make clear. Clark has the most important piece, but only a sub-component of the answer.

The refutation first. As it turns out I am sitting in my office and I am thirsty. But there is no water here. Per Clark, I am essentially a prediction machine. I am driven by my brain whose ultimate purpose is to cancel out discrepancies between what I predict my sensory input to deliver and what it actually delivers. So I look around from my chair and form various low-level (mostly unconscious) predictions about what I will see as I turn my head. I am mostly correct (I have spent many hours here, after all) but I am off in numerous small ways for which I adjust. And I sit. I predict the continued sensation of the chair against my back. And I sit. And I automatically engage more predictions. And I sit. And I sit. And, eventually, as it turns out, I die of thirst.

Why did I just die? Because I became essentially a prediction machine instead of essentially a living survival machine. A prediction machine passively predicts; a survival machine takes action to survive. Is prediction so fundamental to us that it can explain mind and action? I submit, no. It offers no explanation of why, in reality, when I am thirsty I get up from my chair and walk down the hall to get a glass of water. In fact, on its own, it can never explain any action (though it is a critical component of every action). This approach in the worst case leads one to sterilize psychology clean of drives, desires, and motivations to action, a la the “desert landscape” version of Friston et al. (2011), or forces one to somehow tac them on *ad hoc*.

What are living organisms, essentially? This might seems like philosophical grist for generations to come, but biology has a rather simple answer. Living creature do things; inanimate objects do not. We, and all animals across the entire phylogenetic scale, actively control ourselves and select portions of the world around us to advance our success in the environment. And since the origins of life we have relied on exactly one principal to do this.

Three to four billion years ago the first single-celled microorganisms enclosed themselves in a phospholipid membrane which provided a clearly demarcated boundary between that portion of the environment that could be readily controlled and the rest of the world. The advantages of controlling one's immediate environment are enormous. It frees the creature from the oscillations of the external world and allows it to hold fixed, in the face of environmental variability, conditions it can rely on to more easily thrive in the world. This is the core principal of physiology (Michael et al., 2009)^1^; even evolution depends on it. The principle at work has a name. It is called homeostasis. Homeostasis is the active regulation (the control) of some parameter such that if that parameter falls outside its target range the regulatory system will resist that change and recover back to target. Such systems can be fleetingly brief of life-long. Inherent in a living organism, homeostasis has been so successful that it is multiplied thousands of thousands of times deep into every system of the body of an advanced mammal like us.

In general, the greater the number of homeostatic regulatory systems the more evolutionarily advanced the creature and the greater its ability to succeed in diverse conditions. Cold-blooded creatures (ectotherms) like snakes, do not have a homeostatic system for regulating temperature so they are slowed to the point of immobility in cold weather, while an endotherm like you and I can function pretty much the same as we do when we are in warmer climes. That is the advantage of a temperature regulating system that keeps our body temperature within a tight range of variability.

Physiology is the study of the functions of living organisms and every physiological function is commonly understood as a homeostatic system – the density of our bones, the beating of our heart, the blinking of our eyes, the release of adrenaline, the acidity of our stomachs, the firing of our neurons, the rate of cell turnover, the population of digestive bacteria in our guts, the secretion of the amylase enzyme, the levels of our hormones, the concentration of CO_2_ in our blood, an so on. The curious reader can grab any standard physiology text and bone up on a nearly endless list of them.

Every physiological system is now understood as a system that “strives” to maintain control over select biochemical parameters to keep them within a “desired” range… with one rather notable exception in the literature. That exception is the mind. But why should the mind be given the right of exceptional exclusion from the otherwise exception-less rule of physiology? Clearly the brain, the extended nervous system, and its enormous variety of interrelated homeostatic systems cannot be excluded from the natural order. So, unless one takes up the unenviable position of arguing for a division between the brain and mind so sharp as to leave the former in the domain of natural law and the latter outside it, then the mind, too, is a homeostatic system, or more accurately, a collection of homeostatic regulatory control systems (albeit, many quite dynamic or short-lived). Let's call this the Homeostasis Thesis: The mind, like all physiological systems is a homeostatic system (for a more detailed analysis see McBride, 2008/2011).

If the entire central nervous system is a massive collection of homeostatic systems then the cognitive and behavioral activities that supervene over the central nervous system are also homeostatic. And this is easy to see in any of the vast arrays of behavioral patterns we deploy. The behavior of living organisms is directed under its own control. It exerts control through its behavior in order to satisfy its needs, desires, and values^2^. This control system is best understood the way any physiological control system is – as a homeostatic system. There is an unfolding of sensorimotor experiences that I expect as I, e.g., tie my shoe. That temporally extended pattern forms the extended target within which I keep my sensorimotor experiences as I tie my shoe. As I deviate from them, say, by failing to push one lace completely under the other, I adjust and push further to get the shoelace under.

Every homeostatic system has the following features: A regulated parameter; a target goal state; a correction system; a context of regularities the system depends upon; and an evaluation system. The critical piece of any homeostatic system is the error correction system which corrects back toward a target goal state. It is an active process. Of course, on a more passive approach, we could call the goal state the “prediction” (and the correction system “error correction”). This brings us to the punchline you can now guess: Clark's error correction under prediction is only a part (arguably the most important part) of a larger structure at play – the homeostatic system. And error correction under prediction is everywhere we look because each error correction system is part of a homeostatic system and homeostasis is everywhere.

There are advantages to understanding cognition as thoroughly homeostatic instead of thoroughly sprinkled with only the error correction component of the homeostatic model. First, it provides a more complete account. Second, it seamlessly merges with the entire corpus of physiology and biology. Third, it inclines us toward a more accurate account of the purpose of cognition: to increase our active successful engagement of our environment (not passive prediction of it). Fourth, it disinclines us away from the problematic “desert landscape vision” of Friston et al. (2011) where goals, motivations, values, drives, and reward signals are eliminated from our psychological existence en-masse, napalm-style. Fifth, it is inherently an account of activity so, unlike the prediction model, it does not struggle to explain action. Sixth, it is inherently a control system framework so it merges perfectly with more detailed control system interpretations. Finally, all the wonderful low-level neural processing that Clark describes (like evidence that targets/predictions flow downward and error signals flow upward) is accommodated, and perhaps even more naturally explained, under the homeostatic model.

Many background commitments of Clark I am equally supportive of: the possibility of a common underlying neural “computation,” and hence, blurring and interplay, between action and sensation^3^, the importance of feedback loops at lower and higher levels, probabilistic generative modeling, and, the idea supported by Clark that the mind strives to “lower information-theoretic free energy” … or put in my terms: the mind is a self-sustaining system striving for equilibrium against a assaults of internal and external forces. Action is often the shortest course to equilibrium, so thirst, among many other drives, bumps one out of equilibrium, and serves as a motivating force to acquire water.

So, the homeostatic approach is not a rejection of the overwhelming majority of the content of Clark's excellent work, but rather a framework for it. This both streamlines the middle and higher level work and provides a more compelling structure within which to elucidate the lower-level advances in neuroscience he describes.

## Footnotes

^1^They describe nine core principles of physiology and the other eight, arguably, all depend on homeostasis.

^2^A more complete account of how motivations and drives come to attach to certain behavior patterns is discussed in more detail in McBride (2008/2011).

^3^The prediction model has a harder time explaining action; the homeostatic model has a harder time explaining sensation. The mechanics, as Clark and I agree, are similar. With sensation, top-down neural activity is adjusted based on the resultant error (and the prediction, aka the temporary homeostatic target, is modified). With action, there is a larger action goal/target that remains in place while top-down neural activity that connects to motor output is adjusted based on the resultant deviation from expected sensorimotor activity.
